# Isolation and Evaluation of Erinacine A Contents in Mycelia of *Hericium erinaceus* Strains

**DOI:** 10.3390/foods13111649

**Published:** 2024-05-24

**Authors:** Mengchen Liu, Liangliang Liu, Xiaoya Song, Yingjun Zhou, Yuande Peng, Chunliang Xie, Wenbing Gong

**Affiliations:** 1 Institute of Bast Fiber Crops, Chinese Academy of Agricultural Sciences, Changsha 410205, China; mengchenliu0108@163.com (M.L.); liuliangliang@caas.cn (L.L.); zhouyingjun@caas.cn (Y.Z.); ibfcpyd313@126.com (Y.P.); xiechunliang@caas.cn (C.X.); 2 Lishui Academy of Agricultural and Forestry Sciences, Lishui 323000, China; lssyj323@163.com

**Keywords:** lion’s mane mushroom, erinacines, separation and purification, high-speed countercurrent chromatography, wild germplasm resources

## Abstract

*Hericium erinaceus* has long been favored for its remarkable nutritional and health-promoting benefits, and erinacine A is the key component responsible for the neuroprotective properties of *H. erinaceus*. Establishing an efficient method for separating erinacine A from *H. erinaceus* and screening the erinacine A-enriched strains is crucial to maximizing its benefits. Herein, we first reported that high-speed counter current chromatography (HSCCC) is an effective method for separating high-purity erinacine A. Using a two-phase solvent system composed of *n*-hexane/ethyl acetate/methanol/water (4.5:5:4.5:5, *v*/*v*/*v*/*v*), erinacine A with a purity of over 95% was separated. Then, we evaluated the content and yield of erinacine A in the liquid-fermented mycelia of *Hericium* germplasms. Both the content and yield of erinacine A varied greatly among the surveyed strains. The significant effect of the strain on the erinacine A content and yield was revealed by an analysis of variance. The highest erinacine A content and yield were observed in the mycelia of a wild strain HeG, reaching 42.16 mg/g and 358.78 mg/L, which is superior to the current highest outcomes achieved using submerged cultivation. The isolation method established and the strains screened in this study can be beneficial for the scaling up of erinacine A extraction and nutraceutical development to industrial levels.

## 1. Introduction

Mushroom production is ecologically, agriculturally, industrially, and medicinally important, as it converts lignocellulosic agricultural wastes into food, feed, and fertilizer [1]. Mushrooms represent an important and sustainable food source with favorable medicinal properties [2]. Large amounts of nutrients and bioactive components, such as high-quality proteins, polysaccharides, terpenoids, etc., have been isolated from both mycelia and fruiting bodies of mushrooms. Recently, mushroom nutraceuticals have been adopted as dietary supplements to enhance general health and fitness [3]. These environmental and economic benefits demonstrate that mushrooms and their mycelia fit within the UN Sustainable Development Goals (SDGs), which have the aim of eradicating hunger while providing food and nutrition security within a sustainable agricultural system by 2030 [4,5].

*Hericium erinaceus* (Bull.) Pers., also known as lion’s mane mushroom, monkey’s head mushroom, or Yamabushitake, is a widely cultivated edible mushroom with a long history of use in traditional Chinese medicine [6]. Both the mycelia and fruiting bodies of *H. erinaceus* are rich in beneficial metabolites, including polysaccharides, erinacines, hericenones, and ergosterols [7,8,9]. Among all the culinary medicinal mushrooms, the nootropic properties of *H. erinaceus* have been especially well-documented, especially in their use in neuroprotection against neurodegenerative diseases, including Alzheimer’s and Parkinson’s diseases [10,11].

Among the bioactive metabolites of *H. erinaceus*, erinacines are the key components responsible for its neuroprotective effects [12]. To date, dozens of erinacines have been isolated from *H. erinaceus* mycelia. Among them, erinacine A is a well-established compound exhibiting versatile neuroprotective bioactivity [13]. Studies have demonstrated that erinacine A could reduce amyloid deposition, inhibit amyloid β production, and promote neurogenesis, making it a potential candidate for therapeutic use in Alzheimer’s disease [14]. Furthermore, erinacine A has been shown to protect dopaminergic neurons against inflammatory factor-induced cell death, prevent glial cell activation, and enhance memory in neurodegenerative diseases [15,16,17,18].

As erinacine A is a promising candidate for the development of health-promoting agents, there is a growing interest in the separation and purification of erinacine A from *H. erinaceus*. Until now, no erinacines have been commercially available due to the difficulty of isolating pure erinacines from *H. erinaceus* on a large scale. In most studies on erinacines, they were mainly self-prepared by using silica gel column chromatography [19,20,21], which is tedious and laborious. Alternatively, high-speed countercurrent chromatography (HSCCC) has the advantages of a low sample denaturation risk, rapid separation, high recovery, etc., and has been widely used to isolate and purify bioactive compounds from various natural sources [22,23]. In mushrooms, HSCCC has also been used for the isolation and purification of metabolites, including polysaccharides, triterpenoids, and flavonoids [24,25,26,27]. The diversification of HSCCC applications inspired us to investigate its adaptability and potential for the separation and purification of erinacines.

As one of the secondary metabolites, the content of erinacine A has shown marked variation among different strains [8], and was regulated by multiple genes. Therefore, the acquisition of *H. erinaceus* strains rich in erinacine A is essential to maximize its benefits. As the major producer, China harbors abundant germplasm resources of *H. erinaceus* [28]. However, the performance of nutrients and health-promoting compounds, including erinacine A, in *H. erinaceus* germplasm resources has not been evaluated. Herein, we reported the isolation of erinacine A by using HSCCC, and evaluated the contents and yields of erinacine A in germplasm resources of *Hericium* collected from China. This study aimed to isolate high-purity erinacine A by using the HSCCC method and to identify superior *H. erinaceus* strains with a desirable abundance of erinacine A, which could effectively promote the application of erinacine A.

## 2. Materials and Methods

### 2.1. H. erinaceus Strains and Mycelial Growth

In this study, a total of 15 *H. erinaceus* strains, including 4 cultivars, 3 hybrid strains, and 8 wild strains, were collected from China. Furthermore, two *H. coralloides* strains were also collected and analyzed. All the *Hericium* strains were deposited at the Institute of Bast Fiber Crops, Chinese Academy of Agricultural Sciences (Changsha, China).

The mycelial growth of all the *Hericium* strains was evaluated on MEA medium (malt extract agar: 3% malt extract, 0.3% soya peptone, and 1.5% agar). The 5 mm mycelial discs were cut from the advancing margin of seven-day-old pure cultures and placed in the center of a 9.0 cm petri dish filled with 20 mL of MEA medium [29]. The *Hericium* strains were then incubated in the dark at 25 °C with five biological replicates. Mycelial growth rate was calculated as the radial extent of each mycelial colony per day [30].

### 2.2. Preparation of Erinacine A Crude Extract

The lyophilized mycelia of *H. erinaceus* were purchased from Hunan Jinnong Agricultural Technology Co., Ltd. (Changsha, China). Erinacine A standard (purity > 95.0%) was generously provided by Prof. Chengwei Liu (Northeast Forestry University, Harbin, China). Erinacine A extraction was performed as described by [8] with minor modifications. The above-mentioned lyophilized mycelia of *H. erinaceus* were ground to powder, and then 1 kg of the powder was added into 75% ethanol (*w*/*v*: 1/20) and extracted by ultrasonication for 1 h at 50 °C twice. The extract was centrifuged at 8000× *g* for 10 min, then filtered through 0.45 µm microfilters and concentrated by vacuum rotary evaporation at 50 °C. Then, the extract was diluted with deionized water to 50 mL, and mixed with ethyl acetate (*v*/*v* ratio 1/1) in a separatory funnel. After a vigorous shake, the ethyl acetate layer was separated and dried by vacuum rotary evaporation. The dry extract was re-dissolved in ethanol and filtered through 0.45 µm microfilters for HPLC analysis.

### 2.3. Determining the Two-Phase Solvent System

The selection of a suitable two-phase solvent system is essential for successful HSCCC separation. This system would ensure the separation of the target compound based on its partition coefficient (*K* value). To determine the *K* value of the two-phase solvent system, a standardized HPLC technique was used as follows. First, a sufficient amount of the solid sample was diluted in a pre-mixed two-phase solvent, and then shaken vigorously to reach equilibrium. After that, the two phases of the solution were separated, and the resulting residues of each phase were obtained by removing the solvent with a rotary evaporator. Afterwards, the residues were dissolved in 1 mL of methanol and filtrated through a 0.45 μm filter for subsequent HPLC analysis. The *K* value was defined as the ratio of the peak area associated with the target compound in the stationary phase to the peak area in the mobile phase.

### 2.4. Preparation of Two-Phase Solvent System and Sample Solution

In this study, a two-phase solvent system was prepared by mixing *n*-hexane, ethyl acetate, methanol, and water, respectively, in a volume ratio of 4.5:5:4.5:5 to satisfy the separation requirement. Each part of the solvent was mixed and shaken vigorously in a separation funnel, where it was allowed to reach a state of equilibrium at ambient temperature. The upper and lower phases were then clearly separated and ultrasonicated for 30 min to remove any dissolved gases. To prepare the test liquid for HSCCC separation, 200 mg of the crude extract, in dry powder form, was uniformly dispersed in 10 mL of solvent mixture consisting of equal parts of the upper and lower layers (5 mL each) obtained from the two-phase solvent system.

### 2.5. HSCCC Isolation

For HSCCC experiments, TBE-300A HSCCC apparatus was used (Shanghai Tauto Biotech, Shanghai, China). This apparatus contained three PTFE multilayer coil columns in series. The inner diameter of the tubes was 1.6 mm, the total column volume was 280 mL, and a 20 mL sample loop was connected. The revolution radius was 5 cm. The β values of the multilayer coil ranged from 0.5 at the internal terminal to 0.8 at the external terminal. The revolution speed was regulated in a range between 0 and 1000 rpm by a speed controller. This HSCCC apparatus was also equipped with a steady-flow pump, a thermal circulation system, and a UV detector.

Prior to the separation of different samples, the column was initially loaded with the upper phase, which acted as the stationary phase. Following this, the device was rotated at a speed of 950 rpm, and the lower phase, functioning as the mobile phase, was introduced into the column at a flow rate of 2.0 mL/min. When the system reached a hydrodynamic equilibrium, the sample solution was loaded into the system. The temperature was maintained at 25 °C, and the current monitored at 340 nm was manually collected every 5 min according to the chromatography. The collected fractions were appropriately labeled and stored for further HPLC analysis to identify the target compounds.

### 2.6. Quantification of Erinacine A Content in Different Hericium Strains

For all the *Hericium* strains, the same procedure was adopted for mycelium production and erinacine A extraction. Briefly, to prepare the seed cultures, six mycelia discs (5 mm in diameter) were inoculated into 300 mL shaking flasks with 100 mL MYG medium (1% malt extract, 0.1% peptone, 0.1% yeast extract, 2% glucose). After inoculation, the 300 mL shaking flasks were incubated for 10 d at 180 rpm and 25 °C in a shaded incubation shaker. Then, the above seed culture was inoculated (*v*/*v* ratio 1/10) into 1 L sterile MYG medium in 3 L Hinton flasks. Cultivation was carried out in a rotary shaker at 25 °C for 40 days with constant shaking at 180 rpm. After that, the mycelia were harvested by filtration, rinsed with deionized water, freeze-dried, and weighed. Following the above-mentioned procedures, the erinacine A was extracted from the different strains.

To quantify the erinacine A content in different *Hericium* strains, the filtered sample was analyzed using an Agilent 1260 HPLC system equipped with a UV-Vis DAD detector (Agilent Technologies Inc., Santa Clara, CA, USA). Chromatographic separation of the analytes was achieved by using a Supersil AQ-C_18_ column (5 μm, 250 × 4.6 mm). The analysis was performed at 25 °C using an acetonitrile–water (55:45 *v*/*v*) isocratic elution at a flow rate of 1.0 mL/min, and an injection volume of 5 µL. The UV detection was performed at 340 nm. Erinacine A was well separated, with a retention time of 11.2 min. The seven-point calibration curve of erinacine A was obtained using a linear fit, and ranged from 50 μg/mL to 3200 μg/mL (y = 2.6396 x − 138.77), with R^2^ values of 0.999 (Supplementary File, Figure S1). Erinacine A of *Hericium* mycelia was determined by comparing the retention time to that of the standard, and the quantification was conducted by using this calibration curve. All the experiments were performed in triplicate.

### 2.7. Statistical Analysis

All the statistical analyses were performed using SPSS 25.0 (SPSS Inc., Chicago, IL, USA). One-way analysis of variance was used to evaluate the effects of the strains on erinacine A content and yield. Statistical significance was then assessed by multiple comparisons using Duncan’s test (*p* < 0.05). Pearson coefficient analysis was employed to calculate correlations between pairs of surveyed traits.

## 3. Results and Discussion

### 3.1. Selection of the Solvent System

In HSCCC analysis, an appropriate two-phase solvent system is a critical factor in determining the analytical outcome. It is guided by the chemical properties of the target compounds and provides the desired partition coefficient (*K*). An ideal *K* value ranges from 0.5 to 2.0. Smaller *K* values would cause solutes to approach the solvent frontier. Larger *K* values could provide better resolution but may result in dilated and attenuated peaks due to extended elution times [31]. Following these principles, several two-phase solvent systems based on *n*-hexane/ethyl acetate/methanol/water (3:5:3:5, 4:5:4:5, 4.5:5:4.5:5, and 5:5:5:5, *v*/*v*/*v*/*v*) were evaluated. The *K* values of the erinacine A were measured, and the results are summarized in Table 1. The results showed that the compounds were predominantly distributed in the upper phase in the 3:5:3:5 system. Therefore, the ratios of *n*-hexane and methanol were increased to change the polarity of the system. As the ratios increased, the *K* values of the erinacine A decreased to the appropriate range. When the ratio reached 4.5:5:4.5:5, the *K* value of the erinacine A (0.81) was proper for the separation [32]. In *H. erinaceus*, the appropriate HSCCC solvent systems for isoflavone isolation were also selected based on similar *K* values [26]. Finally, the *n*-hexane/ethyl acetate/methanol/water system (4.5:5:4.5:5, *v*/*v*/*v*/*v*) was selected due to its favorable *K* value.

### 3.2. Separation by HSCCC and Identification of Erinacine A

**Figure 1:**
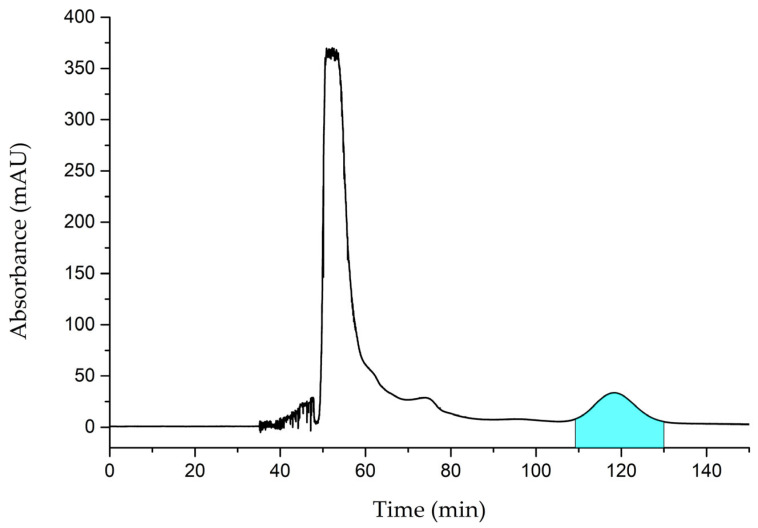
The HSCCC separation chromatogram of extract. The blue area was collected for the HPLC analysis.

**Figure 2:**
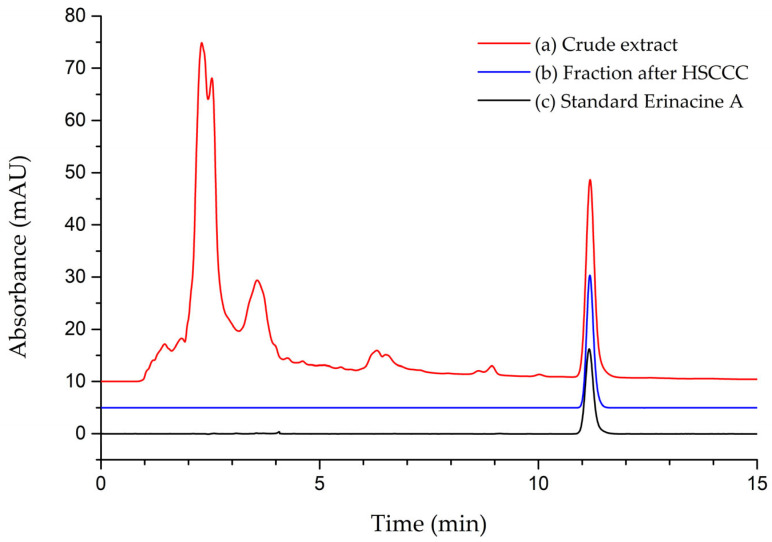
The HPLC analysis of extract crude of HeG sample (**a**), fraction after HSCCC (**b**) and standard erinacine A (**c**).

Using the established parameters, HSCCC separation was performed, and the representative HSCCC chromatogram of the extract is shown in Figure 1. The analysis lasted approximately 150 min and resulted in the isolation of erinacine A. The target compound eluted at about 120 min, and the fraction from 110 min to 130 min was collected for further HPLC analysis. The corresponding chromatographic profiles of the collected target fraction are illustrated in Figure 2. The chromatograms of the extract and standard erinacine A are also presented for comparison. In the HPLC analysis, the peak of erinacine A appeared at 11 min. Both standard erinacine A and the collected fraction showed only one pure peak in all plots, and the retention times of the peaks were the same as that of extract. By comparing with the standard, it was seen that the UV spectrum of the isolated erinacine A was the same as those of the standard. Ultimately, a high yield of erinacine A (≥98%) was obtained from the extract following the HSCCC separation. Moreover, the purity of the fraction was over 95%, as determined by utilizing the peak area normalization method. Initially, erinacine A was isolated by repeated silica gel chromatography [33]. After that, most of the erinacines were also isolated using silica gel chromatography [19,20,21,34], and there were few reports of isolating erinacines using other methods. Compared to column chromatography, HSCCC showed advantages in requiring fewer cycles for high purity products, less solvent usage, higher efficiency, and less labor [35]. These results demonstrated that the isolation of erinacine A with HSCCC was well completed.

### 3.3. Mycelial Growth of Hericium Strains

**Figure 3:**
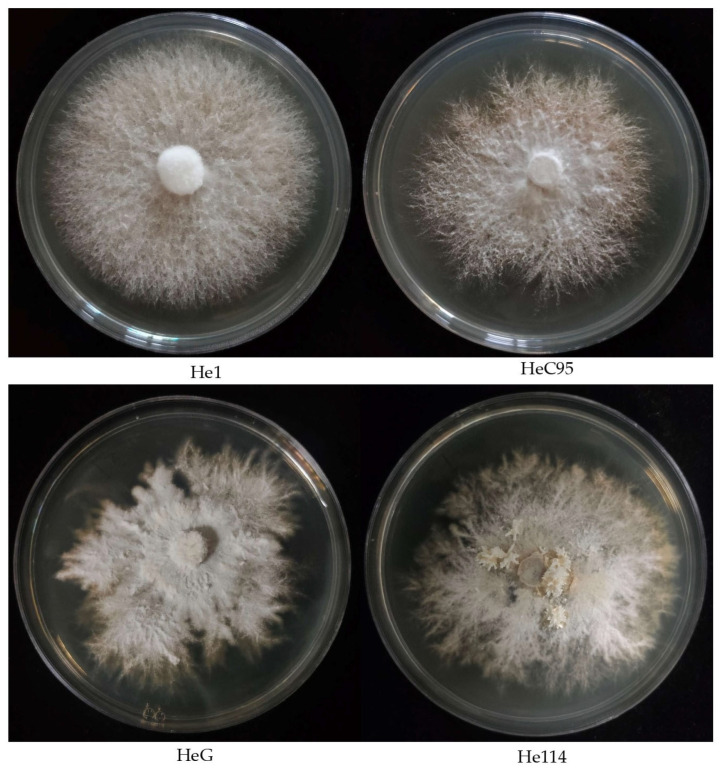
Typical colony morphologies of the investigated *Hericium* strains.

In mushrooms, mycelial growth plays a crucial role in substrate colonization, disease resistance, and yield, and is also of great significance for metabolite productions, such as erinacine A [30,36]. In this study, the mycelial growth rates of the investigated *Hericium* strains on MEA medium differed significantly, ranging from 2.10 mm/d to 4.31 mm/d (Table 2). He1 collected from Sichuan Province showed the fastest mycelial growth. In addition, the mycelial growth rates of the commercially cultivated strains were significantly higher than those of the wild strains (3.85 mm/d vs. 2.74 mm/d, *p* < 0.01). The colony morphologies of the investigated *Hericium* strains were also markedly different (Figure 3). The colonies of all four cultivated strains on the MEA medium were circular, whereas several wild strains (such as HeG, HeV, and HeR) exhibited irregular colony morphologies (Figure 3).

The mycelial growth rates of the two wild *H. coralloides* strains He61 and He114 were 2.12 mm/d and 2.49 mm/d, respectively, lower than most of the *H. erinaceus* strains. In addition, primordia formations were observed for He61 and He114 on the MEA medium, indicating that they were prone to fruiting (Figure 3).

Fungi form colonies or mycelia that consist of a large interconnected network of hyphae. Mycelial growth, characterized by an invasive growth rate and its colony morphology, is mainly affected by the nutritional compositions of the medium, culture condition, and complex gene regulation [30,37,38]. The complexity and the heterogeneity of hyphae, such as the apical expansion, arrangements, and branching angle, may influence the colony macroscopy and cause irregular morphology [38]. In *Hericium*, differences in mycelial growth and colony morphology related to strain genotype and culture conditions were observed [29,39,40]. In this study, the differences in mycelial growth and colony morphology between the surveyed *Hericium* strains may be largely due to due to differences in their genetic backgrounds. A relationship between the mycelial morphology and growth rate was observed in *Pleurotus* spp. [41]. In this study, although there was no clear correlation between the mycelial growth rate and colony morphology, the *H. erinaceus* strains with irregular colonies showed slower mycelial growth. This may be due to the fact that mycelial growth was one of the improvement targets during *H. erinaceus* breeding, and the fast-growing strains with circular colonies were prone to be selected.

### 3.4. Erinacine A in the Mycelia of Hericium Strains

For most mushrooms, the cycle from mycelium colonization to fruiting body formation was time-consuming and laborious. The cultivation of mycelia was the convenient approach to obtain fungal biomass and the bioactive metabolites of interest [36]. Since erinacines were mainly derived from *H. erinaceus* mycelia [10], we evaluated the content and yield of erinacine A in the liquid-fermented mycelia of the surveyed strains. After fermentation, the weight of the lyophilized mycelia was recorded to evaluate the mycelial biomass. As shown in Table 2, the mycelial biomasses of these 17 *Hericium* strains varied from 6.00 g/L to 11.99 g/L. Among them, He61 and HeC95 had a relatively higher production of mycelial biomass (more than 10.0 g/L).

The erinacine A content varied greatly among 17 *Hericium* strains, ranging from 0.23 mg/g to 42.16 mg/g (Table 2). Analysis of variance indicated a significant effect of the genotype (strain) on the erinacine A content (*p* < 0.01, Table 3). Most of the surveyed strains contained low erinacine A. Interestingly, the contents of erinacine A in six *H. erinaceus* strains (HeG, HeC9, HeT, He80, He95, and He911) were more than 1.0 mg/g. Notably, the content of erinacine A in HeG and HeC9 was up to 42.16 mg/g and 21.15 mg/g, respectively. The yield of erinacine A among the surveyed strains during submerged fermentation was determined to range from 1.77 mg/L to 358.78 mg/L. Relatively, the variation range of the mycelial biomass among the surveyed strains was less than that of the erinacine A content (Table 2). Therefore, the content of erinacine A contributed more to the variation in the erinacine A yield. For the two *H. coralloides* strains, He61 and He114 both showed subpar performances in their content and yield of erinacine A. Further, we also analyzed the relationships of mycelial growth rates, mycelial biomass, and erinacine A content and yield. The content of erinacine A was positively correlated to its yield (*p* < 0.01). There was no significant correlation between the mycelial biomass and erinacine A content.

The low productivity of erinacine A has hindered its development and application as a dietary supplement. In the past, great efforts have been made to increase the productivity of erinacine A [8,21,42,43]. Using submerged cultivation, the highest content and yield of erinacine A were reported to be 14.44 mg/g and 225 mg/L, obtained by optimizing the culture medium composition [21,43]. In addition, Cheng et al. [8] reported that solid-state cultivation could significantly increase the content of erinacine A, reaching 165.36 mg/g. At present, there was no literature focusing on the comparison of erinacine A contents in different *H. erinaceus* germplasm resources, or screening the high-content strains or cultivars. Herein, the erinacine A contents sharply differed in the mycelia of different *H. erinaceus* strains. In terms of both erinacine A content and yield, HeG exhibited a superior performance to the current highest outcomes using submerged cultivation, but a lower performance than the results of solid-state cultivation.

### 3.5. Identification of Superior Strains

Currently, the selection and breeding of elite strains with enhanced nutrients and health-promoting compounds is one of the main aims of the mushroom industry [44]. Germplasm resources provide the basic raw material and a reservoir of genes for breeding [45], so the characterization and evaluation of germplasm resources is essential for any breeding program, including edible mushrooms. To date, most studies have focused on estimating the performance of important agronomic traits (such as yield and disease resistance) in mushroom germplasms [46,47]. In *H. erinaceus*, the performance of nutrients and health-promoting compounds in germplasm resources has not been evaluated.

In the present study, the results indicated that the wild germplasm of *H. erinaceus* showed a wide range of variation of erinacine A content. A few strains performed well and may contain superior alleles that regulate the accumulation of erinacine A. In particular, HeG (a wild strain collected from Jiaohe, China), although ordinary in mycelial growth, showed extremely superior in performance both in terms of erinacine A contents and yield, making it promising for breeding erinacine A-enriched cultivars. The hybrid strain HeC9 was generated by the mating of two monokaryons obtained from HeCS and He911, respectively [48]. HeC9 showed a super-parent advantage in terms of erinacine A content and yield, and was also a good candidate for erinacine A production. The development of elite strains and the optimization of fermentation processes were critical for enhancing the productivity of the metabolite of interest [49]. Our results indicated that erinacine A content was the key indicator for elite strain screening to improve its production. HeG and HeC9 exhibited excellent performances and promising application prospects in the development of erinacine A-rich nutraceuticals. Here, these promising strains were identified by evaluating only a handful of *H. erinaceus* strains. China harbors unique and abundant germplasm resources of wild *H. erinaceus* [28,50], and further screening is promising for the identification of better strains.

## 4. Conclusions

For the first time, we efficiently isolated erinacine A using the HSCCC method, evaluated the content and yield of erinacine A in the germplasm resources of *Hericium*, and identified superior strains with desirable performance. Under the optimized two-phase solvent system composed of *n*-hexane/ethyl acetate/methanol/water (4.5:5:4.5:5, *v*/*v*/*v*/*v*), erinacine A was successfully isolated from the extract within 140 min of separation, and the purity of the product reached 95%. Both for content and yield, erinacine A varied greatly among different strains. A wild *H. erinaceus* strain, HeG, exhibited an excellent performance in terms of erinacine A content, and showed great promise in the development of nutraceuticals. The reported separation method could promote the application of erinacine A and expand the related biological research. Our results indicated that the screening of wild strains with excellent performance is an important way to increase the productivity of functional ingredients in mushrooms, and could overcome some potential technological bottlenecks during the product manufacturing process.

## Figures and Tables

**Table 1:** *K* values of erinacine A.

No.	*n*-Hexane	Ethyl Acetate	Methanol	Water	*K* Value
1	3	5	3	5	4.84
2	4	5	4	5	1.19
3	4.5	5	4.5	5	0.81
4	5	5	5	5	0.51

**Table 2:** Erinacine A content and yield in mycelia of the surveyed *Hericium* strains.

Strain	Type	Origin	Mycelial Growth Rate (mm/d)	Mycelial Biomass (g/L)	Erinacine A Content (mg/g)	Erinacine A Yield (mg/L)
He1	Cultivar	Sichuan	4.31 ± 0.173	9.76	0.34 ± 0.004	3.31 ± 0.040
He911	Cultivar	Jiangsu	3.33 ± 0.016	7.03	1.89 ± 0.005	13.28 ± 0.035
HeCS	Cultivar	Changshan, Zhejiang	3.94 ± 0.168	9.52	0.44 ± 0.004	4.22 ± 0.038
HeJ	Cultivar	Sichuan	3.81 ± 0.382	9.23	0.23 ± 0.013	2.09 ± 0.121
HeC95	Hybrid strain	CS-5*911-4	3.57 ± 0.067	10.57	2.10 ± 0.007	22.23 ± 0.079
HeC9	Hybrid strain	CS-4*911-4	3.03 ± 0.242	6.99	21.15 ± 0.098	147.87 ± 0.686
He92	Hybrid strain	Lishui, Zhejiang	2.10 ± 0.034	6.00	0.30 ± 0.002	1.77 ± 0.010
He34	Wild strain	Wangqing County, Jilin	2.18 ± 0.099	9.66	0.29 ± 0.001	2.77 ± 0.014
He80	Wild strain	Khingan Mountains, Heilongjiang	3.43 ± 0.100	8.45	3.66 ± 0.015	30.92 ± 0.125
He89	Wild strain	Dongning County, Jilin	3.29 ± 0.089	7.93	0.38 ± 0.005	3.04 ± 0.041
HeG	Wild strain	Jiaohe, Jilin	2.27 ± 0.185	8.51	42.16 ± 0.181	358.78 ± 1.537
HeR	Wild strain	Yichun, Heilongjiang	2.89 ± 0.096	8.79	0.60 ± 0.002	5.26 ± 0.020
HeT	Wild strain	Yichun, Heilongjiang	3.03 ± 0.195	9.70	4.07 ± 0.024	39.47 ± 0.238
HeU	Wild strain	Yichun, Heilongjiang	3.42 ± 0.227	7.69	0.50 ± 0.014	3.88 ± 0.107
HeV	Wild strain	Yichun, Heilongjiang	2.30 ± 0.159	7.21	0.33 ± 0.001	2.38 ± 0.008
He61 ^#^	Wild strain	Khingan Mountains, Heilongjiang	2.12 ± 0.109	11.99	0.30 ± 0.001	3.60 ± 0.006
He114 ^#^	Wild strain	Khingan Mountains, Heilongjiang	2.49 ± 0.155	8.28	0.39 ± 0.001	3.20 ± 0.003

Note: ^#^ He61 and He114 were two *H. coralloides* strains. * HeC95 was generated by mating between CS-5 and 911-4 while, HeC9 was generated by mating between CS-4 and 911-4.

**Table 3:** Analysis of variance of content and yield of erinacine in *Hericium* strains.

	Source of Variation	Sum of Squares	Degree of Freedom	Mean Squares	F Value	*p* Value
Erinacine A content	Between groups	5688.739	16	355.546	138,662.199	<0.001
	Within groups	0.087	34	0.003		
	Total	5688.826	50			
Erinacine A yield	Between groups	387,580.136	16	24,223.759	139,967.804	<0.001
	Within groups	5.884	34	0.173		
	Total	387,586.021	50			

## Data Availability

The original contributions presented in the study are included in the article and supplementary materials, further inquiries can be directed to the corresponding author.

## Supplementary Materials

The following supporting information can be downloaded at: https://www.mdpi.com/article/10.3390/foods13111649/s1, Figure S1: The seven-point calibration curve of erinacine A.
