# Supplementary figures and images for: Isolation and Evaluation of Erinacine A Contents in Mycelia of Hericium erinaceus Strains
Source: Foods. 2024 May 24;13(11):1649. doi: 10.3390/foods13111649 (PMC11172171; doi:10.3390/foods13111649)

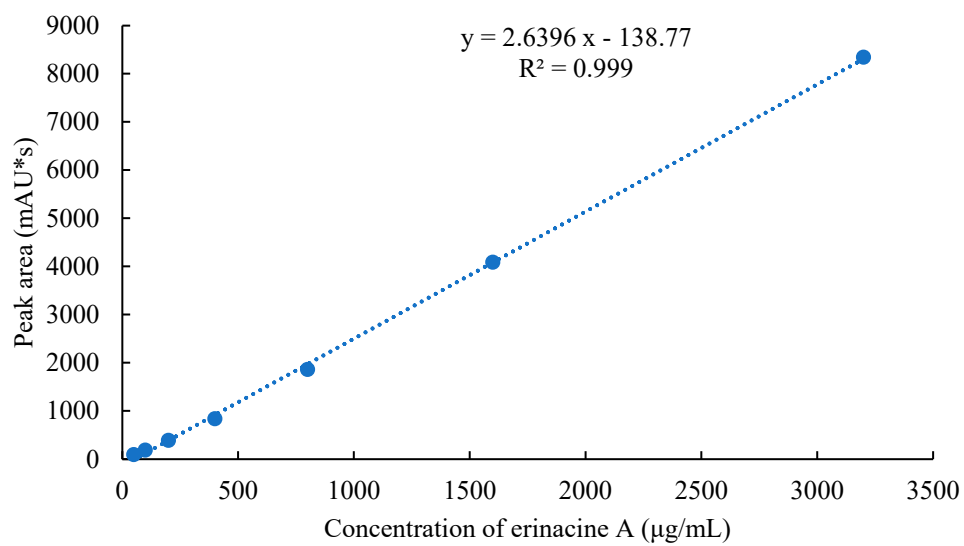

**Figure S1. The seven-point calibration curve of erinacine A.**

Supplement: Supplementary file 1 [file foods-13-01649-s001.zip › foods-3011820-supplementary.pdf]
